# Detection of Hydraulic Phenomena in Francis Turbines with Different Sensors

**DOI:** 10.3390/s19184053

**Published:** 2019-09-19

**Authors:** David Valentín, Alexandre Presas, Carme Valero, Mònica Egusquiza, Eduard Egusquiza

**Affiliations:** Center for Industrial Diagnostics and Fluid Dynamics (CDIF), Universitat Politècnica de Catalunya (UPC), Av. Diagonal, 647, ETSEIB, 08028, Barcelona, Spain; alexandre.presas@upc.edu (A.P.); m.del.carmen.valero@upc.edu (C.V.); monica.egusquiza@upc.edu (M.E.); eduard.egusquiza@upc.edu (E.E.)

**Keywords:** hydropower, Francis turbine, sensors, monitoring, vibration, detection

## Abstract

Nowadays, hydropower is demanded to provide flexibility and fast response into the electrical grid in order to compensate the non-constant electricity generation of other renewable sources. Hydraulic turbines are therefore demanded to work under off-design conditions more frequently, where different complex hydraulic phenomena appear, affecting the machine stability as well as reducing the useful life of its components. Hence, it is desirable to detect in real-time these hydraulic phenomena to assess the operation of the machine. In this paper, a large medium-head Francis turbine was selected for this purpose. This prototype is instrumented with several sensors such as accelerometers, proximity probes, strain gauges, pressure sensors and a microphone. Results presented in this paper permit knowing which hydraulic phenomenon is detected with every sensor and which signal analysis technique is necessary to use. With this information, monitoring systems can be optimized with the most convenient sensors, locations and signal analysis techniques.

## 1. Introduction

The role of hydropower has changed in the last years with the massive entrance of other renewable sources in the energy market [1,2]. Nowadays, hydraulic turbines are demanded to work in their whole operating range requiring a fast response between load changes. Unlike Pelton or Kaplan turbines that present a good performance in their whole operating range, Francis turbines are designed to operate at a certain head and discharge where they present their maximum efficiency. The operation under design head and discharge is called best efficiency point (BEP). When Francis turbines operate out of design conditions their efficiency drops and different hydraulic phenomena appear endangering the stability of the machine, reducing the useful lifetime of the turbine components and therefore increasing maintenance and reparation costs.

**Figure 1:**
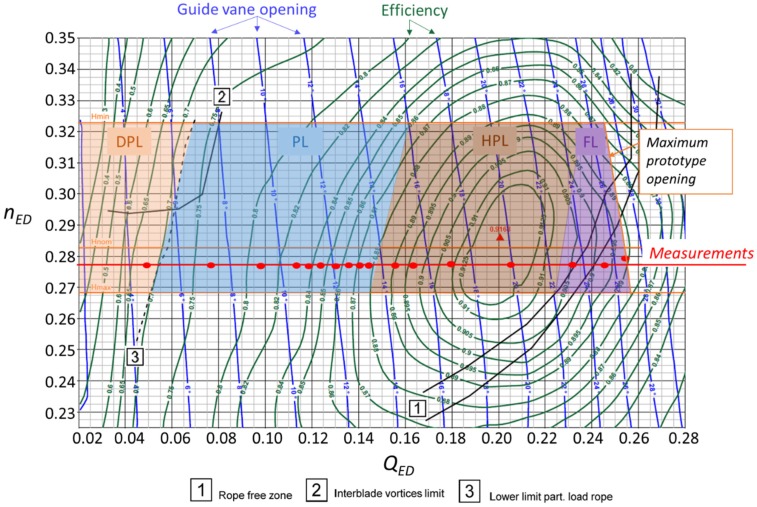
Hill chart of the selected prototype. Operating regimes highlighted in the working range of the prototype.

The operating range of Francis turbines is given by their characteristic hill chart [3] (see Figure 1). In the hill chart, efficiency isolines are shown for the different head and discharge ranges where every Francis turbine unit can operate. Those hill charts are usually generated using reduced-scale models following the IEC (International Electrotechnical Commission) standard [4]. In some hill charts, information about the hydraulic phenomena occurring in every zone is included according to the reduced-scaled model results. However, those phenomena are not always happening exactly in the same way in the prototype than in the reduced-scale model and therefore measurements in the prototype are always required [5,6].

Different operating regimes are usually defined in Francis turbines according to the flow rate passing through the turbine. In every regime a different hydraulic phenomenon appears, every one of a different nature. Cavitation, turbulence or vortex rope are some of the hydraulic phenomena that usually appear in Francis turbines. In addition, those phenomena can lead to hydro-acoustic or mechanical resonances [7,8,9]. The flow-rate is easily controllable in Francis turbines by changing the angle of the guide vanes, however, the head is given by upstream and downstream water levels. Depending on the head, every hydraulic phenomenon in every operating regime can be of a different magnitude [10], and therefore they have to be detected in order to assess their impact on the machine operation and the useful life of the turbine components.

Monitoring systems [11,12,13] are usually installed in hydraulic power plants with the objective of ensuring a safe operation of the machine, performing predictive maintenance and detecting possible failures [14,15,16]. Those monitoring systems are usually based on measuring vibration in the stationary parts of the machine, especially in the bearings, the shaft displacement or oil temperatures. Overall or band levels of vibrations or shaft orbits are normally used for this purpose. However, monitoring systems could be used to detect and identify in real-time the different hydraulic phenomena that appear in Francis turbines, helping to select the best operating condition for the machine depending on the requirements of the electricity generation market at every moment. For this, as a first step, a study of how and which phenomenon is detected with the different sensors is necessary.

In this paper, the detection of the different hydraulic phenomena appearing in a Francis turbine is studied in detail. For this, a large Francis turbine prototype (444 MW of rated power) is selected. Different type of sensors are located in different parts of the turbine in order to see how and which hydraulic phenomena are detected in each one. Accelerometers, proximity probes, strain gauges, pressure sensors and a microphone are installed in both stationary and rotating parts of the machine. In addition, different detection techniques are used for every sensor. As a result, a summary table including which sensor detects every phenomenon, and with which detection technique, is presented. The results obtained in this paper permit improving and optimizing the actual monitoring systems installed in hydraulic power plants.

## 2. Operating Regimes in Francis Turbines

Hill charts are usually generated using reduced-scale models. With reduced-scale models the hydraulic efficiency can be extracted with good accuracy, however some points are not completely transposable from the model to the prototype, as for example, in the case of some hydraulic or mechanical resonances. In order to transpose from model to prototype, the discharge is dimensionless with the discharge factor *Q_ED_* and the head with the speed factor *n_ED_*. The definition of those parameters is found in Equations (1) and (2), where *Q* is the discharge, *D* is the diameter of the runner, *n* is the rotating speed and *E* = *gH* where *H* is the turbine head.

(1) $$Q_{ED}=\frac{Q}{D^{2}\sqrt{E}}$$

(2) $$n_{ED}=\frac{nD}{\sqrt{E}}$$

The hill chart for the selected prototype is shown in Figure 1. In this case, this hill chart was generated in the closed-loop PF3 test rig of the EPFL (École polytechnique fédérale de Lausanne) Laboratory for Hydraulic Machines. The different operating regimes for this Francis turbine are highlighted in the hill chart. There are four different regimes: deep part load (DPL), part load (PL), high part load (HPL) and full load (FL). The main hydraulic phenomena that can be found in any of them are described in the following subsections according to the literature. The BEP is marked in the figure with a red triangle and it is located in the transition between the HPL and FL.

### 2.1. Deep Part Load

This operating regime is characterized by very low discharge. It is found normally below 0.3 times the rated power (P/P_rated_ < 0.3). In this situation, the main hydraulic phenomena occurring in the Francis turbine has a stochastic nature, where turbulence plays an important role, especially in the zone of the draft tube. This stochastic excitation is able to excite wide ranges of frequency, and therefore to excite the natural frequencies of the runner or other turbine components [17,18]. In addition, at certain heads, normally lower than the rated head, interblade vortices appear. Those vortices take place in the runner channels, starting from the crown, decreasing the pressure and producing cavitation. Yamamoto et al. [19,20] studied this phenomenon for the reduced-scale model of the Francis turbine selected for this study.

### 2.2. Part Load

The PL regime is usually defined between 0.3 and 0.6 times the rated power (0.3 < P/P_rated_ < 0.6). For these discharge conditions, the flow at the outlet of the runner presents a swirl that is able to form a cavitating core with a spiral shape in the draft tube, called the vortex rope. This vortex rope presents a frequency precession of about 0.25–0.35 the runner rotating frequency. This phenomenon induces pressure fluctuations in the draft tube cone at the precession frequency and it can be decomposed in two different components: the asynchronous and the synchronous. The asynchronous component corresponds to a rotation pressure pattern in the draft tube and the synchronous component is an axial component that is propagated to the entire hydraulic circuit. The frequency of the vortex rope is dependent on the discharge. If its frequency coincides with a hydro-acoustic natural frequency of any part of the hydraulic circuit, resonance occurs, amplifying substantially the pressure amplitudes. This situation is known as PL resonance and it is able to cause dangerous power swings in the machine [21,22]. The operation at PL resonance is undesirable and should be avoided for as long as possible. Favrel et al. [7,23,24,25,26] studied this phenomenon in detail for the reduced-scale model of the Francis turbine selected for this study.

### 2.3. High Part Load

The HPL regime is defined between 0.6 and 1 times the rated power (0.6 < P/P_rated_ < 1). The vortex rope still exists in this regime, but its intensity has decreased a lot in comparison with the PL operation. In this operation, there are some cases where the HPL vortex rope presents a frequency higher than the runner rotating frequency *f_f_*. In addition, this operation is very near the BEP of the machine, therefore, the main dominant hydraulic phenomenon takin place in this regime is the rotor stator interaction (RSI). 

The RSI is the most important periodic excitation in Francis turbines and it is given by the interaction of the rotating blades of the runner and the stationary blades of the guide vanes [27,28,29]. The pressure field in the gap between blades and guide vanes can be described as the superposition of all the combinations of *m*, *n* (Equation (3)):

(3) $$P_{m,n}\left(\theta ,t\right)=A_{m,n}\cdot \cos\left(mZ_{v}\theta_{s}+\psi_{m}\right)\cdot \cos\left(nZ_{b}\theta_{r}+\psi_{n}\right)$$

where *θ* is the angular coordinate (index *s* is for the stationary coordinate and *r* for the rotating coordinate); *Z_v_* is the number of guide vanes and *Z_b_* the number of runner blades; *ψ* is an angle offset; and *m*, *n* are integer numbers (1, 2, …, ∞) that represent the order of harmonic. Therefore, RSI excitation is a sum of sinusoidal waves at different frequencies with different amplitudes. From the rotating frame, these frequencies are calculated as in Equation (4) and from the stationary frame as in Equation (5). The shape of the wave (*k_m,n_*) associated with each *f_v_,_m_* or *f_b_,_n_* is a combination of the *n* harmonics of *Z_b_* and the *m* harmonics of *Z_v_* (Equation (6)).

(4) $$f_{v,m}=m\cdot Z_{v}\cdot f_{f}$$

(5) $$f_{b,n}=n\cdot Z_{b}\cdot f_{f}$$

(6) $$k_{m,n}=nZ_{b}-mZ_{v}$$

The RSI occurs at all operating regimes of the Francis turbine, but its amplitude (*A_m,n_*) depends on several parameters like the head, operating point, design of the machine and order of harmonics (*m*, *n*).

### 2.4. Full Load

The operation over the rated power (P/P_rated_ > 1) is called full load operation. At this regime another type of vortex rope appears in the runner outlet. In this case this vortex rope is axially centered in the runner cone. The frequency of this vortex rope is normally in the same range as the PL vortex rope (below *f_f_*). For certain discharge and head conditions, the system excites itself at one of its hydro-acoustic natural frequencies and it becomes unstable. This is why this phenomenon is also called self-excited vortex rope or overload instability. At this moment, huge pressure pulsations are originated due to the collapse of the cavitating volume of the vortex rope, which also leads to power swing problems. A detailed study of the physical mechanisms by which the self-excited oscillations are sustained was done by Muller et al. [30,31,32]. Those studies are again carried out with the reduced-scale model of the prototype of study in this paper.

## 3. Experimental Investigation

### 3.1. Prototype Characteristics

A large medium-head Francis turbine (P_rated_ = 444 MW) was selected for this study. This runner of this Francis turbine has 16 blades (*Z_b_* = 16) and a specific speed (ns) of 46. The study of the dynamic behavior of this turbine is part of the collaborative European Project Hyperbole (FP7-ENERGY-2013-1) [33]. The rotating speed of the machine is 128.6 rpm (*f_f_* = 2.14 Hz). There are 20 guide vanes (*Z_v_* = 20), two radial bearings (one in the turbine side and the other in the generator side) and one thrust bearing in the generator side. Thanks to an overhaul in the power plant, the machine was accessible to install several sensors in the rotating parts and in the stationary parts. 

### 3.2. Instrumentation

A total of 67 sensors were installed in the machine for the study. Ten pressure sensors were distributed in the hydraulic circuit including a draft tube, spiral casing and penstock, and eight more were flush-mounted in the runner blades. The runner was instrumented with 24 strain gauges in two different blades. Sixteen accelerometers were installed in the bearings, head cover, guide vanes, spiral casing, draft tube and rotating with the shaft. Four proximity probes were used to measure the shaft displacement in the turbine and generator bearings. The mechanical torque in the shaft was also measured with strain gauges. Electrical parameters such as power, voltage and current were also obtained simultaneously. In addition, one microphone (Bruel & Kjaer Type 4958, 20 kHz maximum frequency) was located near the draft tube wall. Further information about the sensor types and location can be found in [13,17]. All locations and sensors were selected in order to detect the maximum hydraulic phenomena as possible and to understand the dynamic behavior of the unit. Most of the locations are normally used for monitoring in hydraulic turbines (such as the accelerometers in the bearings) and others (like accelerometers in guide vanes, spiral casing and draft tube walls or microphones) are here introduced in this paper to improve those monitoring systems.

**Figure 2:**
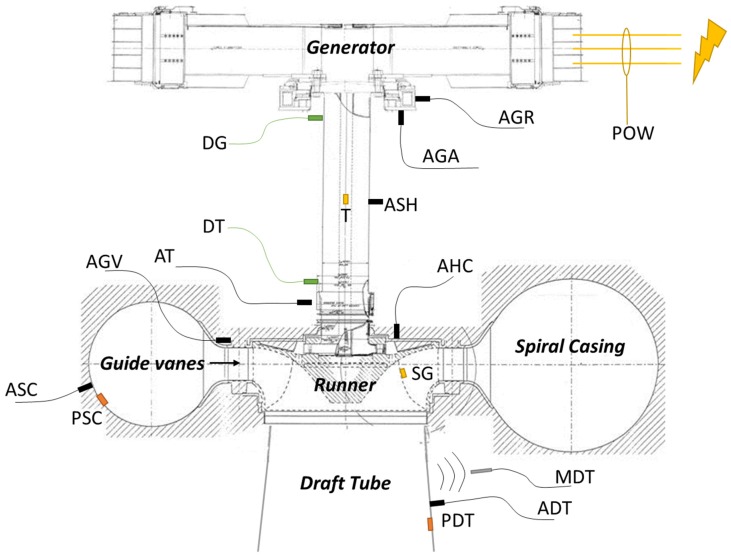
Sensors used for this study.

The sensors were connected to a distributed acquisition system based on 6 modules of 12 channels Bruel & Kjaer LAN XI Type 3053. The signal of the sensors located in the rotating frame was transmitted to the acquisition system by means of a telemetry system. Only one sensor per location and type has been selected for this study, reducing the number of sensors to compare to 16. The sensors selected are shown in Figure 2 and their nomenclature in Table 1. The acquisition frequency for every sensor is also included in Table 1. The maximum frequency of analysis for every sensor is therefore dependent on their acquisition frequency shown in Table 1. Some sensors were acquired at low frequency (4096 Hz) and others at high frequency (65536 Hz) in order to perform demodulation analysis. The microphone acquisition frequency was selected as the same as the accelerometers, but it was analyzed at a maximum frequency of 20 kHz according to its specifications. The accelerometer and the strain gauge in the shaft were acquired at other frequencies due to hardware restrictions.

### 3.3. Testing Procedure

The turbine was operated in its whole operating range. Different points in every regime were selected to study the behavior of the machine. Those points are shown in Figure 1. The machine was working at those points for about 5 minutes in each one in order to have steady conditions. The output power was controlled to ensure the steady condition. The head for all of the measurements were maintained constant, except for the last point in the full load regime, where the head was decreased a little bit in order to reach the overload instability.

## 4. Signal Analysis

Three different signal analysis techniques were used in this paper to detect the different phenomena in every sensor and every operating point: fast Fourier transform (FFT), root mean square (RMS) values and demodulation analysis. For the FFT analysis, a piece of time signal of 60 s was used without performing any average. RMS values were also calculated from the same 60 s signal but applying different bandpass frequency filters to detect the different phenomena. Finally, the demodulation analysis was performed by applying an FFT to the absolute value of the Hilbert transform of 20 s of high-frequency signals filtered in different high-frequency ranges [34,35]. To know which the best high-frequency ranges were to detect every phenomenon, several analyses at different ranges of 2 kHz width were carried out.

## 5. Results

The results obtained are presented in this section and they have been divided according to the detection technique used (FFT, Section 5.1; RMS, Section 5.2; demodulation; Section 5.3). In addition, a table summarizing the detection of every phenomenon with all of the sensors is included in Section 5.4.

### 5.1. FFT

Most hydraulic phenomena occurring in Francis turbines are periodic and related with to the rotating frequency (*f_f_*). Therefore, it is useful to show the results in frequency normalized with the rotating frequency. This is called reduced frequency (reduced frequency = frequency/*f_f_*). 

#### 5.1.1. Low Frequency

**Figure 3:**
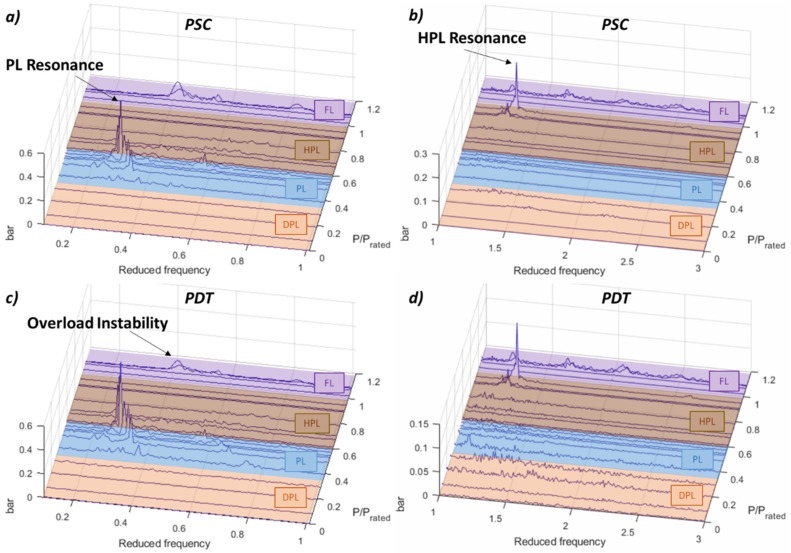
Fast Fourier transform (FFT) waterfall of the pressure sensors. (**a**) PSC 0.1–1 *f_f_*. (**b**) PSC 1–3 *f_f_*. (**c**) PDT 0.1–1 *f_f_*. (**d**) PDT 1–3 *f_f_*_._

Analyzing the low frequency of the pressure sensors, the main hydraulic phenomena related to the vortex rope and hydro-acoustic resonances can be detected. Figure 3 shows the FFT of every operating condition in the low-frequency range (below 3 times *f_f_*). The vortex rope in the PL regime is clearly detected at frequencies between 0.2–0.4 times *f_f_*. Mainly the synchronous component of the vortex rope is detected in the peaks. In the HPL, a resonance is detected at about P/P_rated_ = 0.9 at 1.3 times the *f_f_*. Additionally, the overload instability appears in the FL regime at P/P_rated_ = 1.09.

**Figure 4:**
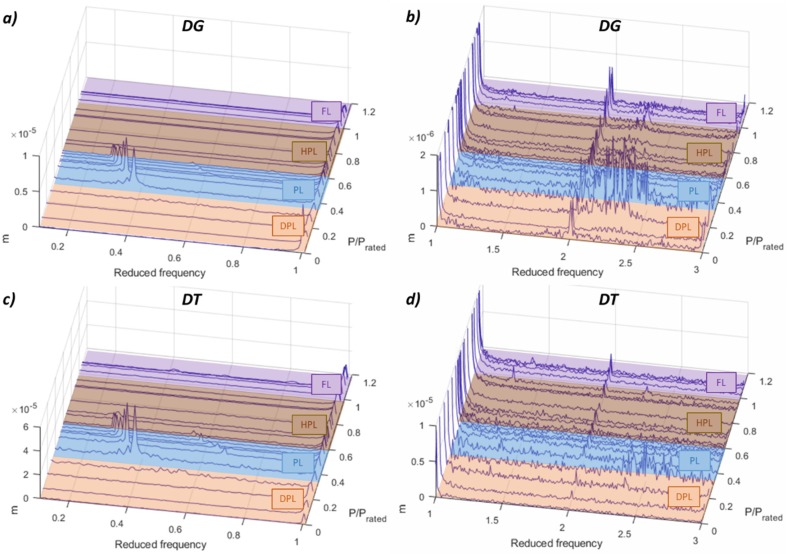
FFT waterfall of the proximity probes. (**a**) DG 0.1–1 *f_f_*. (**b**) DG 1–3 *f_f_*. (**c**) DT 0.1–1 *f_f_*. (**d**) DT 1–3 *f_f_*.

However, observing the results for the proximity probes (see Figure 4), only the asynchronous part of the vortex rope is detected. This is because they are measuring the radial displacement of the shaft, and therefore they only detect the phenomena that cause abnormal radial displacement in the shaft, as it is the case of the asynchronous component of the vortex rope. The PL and HPL resonances as well as the overload instability are not detected with those sensors. Comparing with the rest of the sensors, the proximity probes are the only ones that can detect clearly this asynchronous component of the vortex rope.

**Figure 5:**
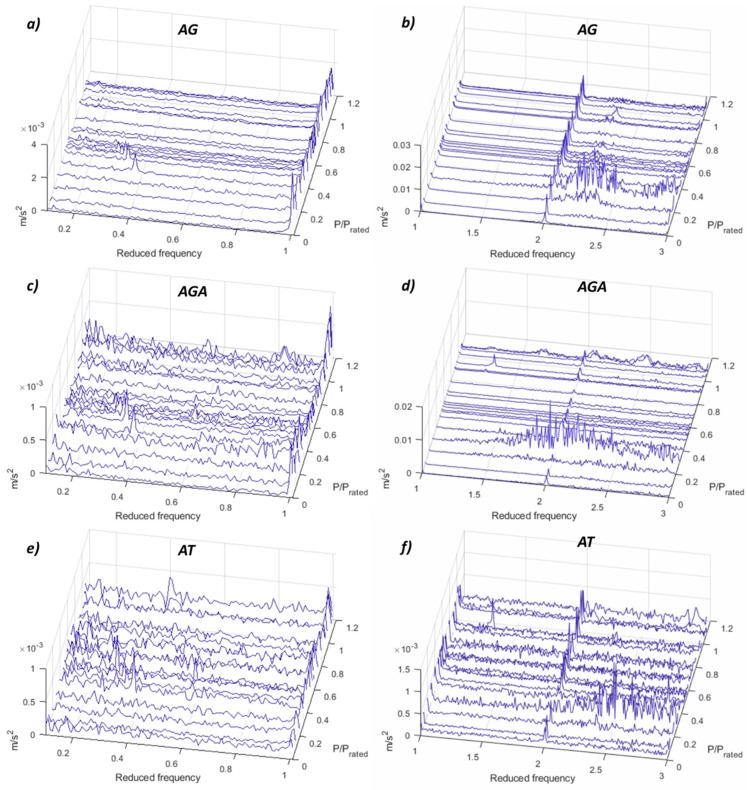
FFT waterfall of the accelerometers in the bearings. (**a**) AG 0.1–1 *f_f_*. (**b**) AG 1–3 *f_f_*. (**c**) AGA 0.1–1 *f_f_*. (**d**) AGA 1–3 *f_f_*. (**e**) AT 0.1–1 *f_f_*. (**f**) AT 1–3 *f_f_*.

The vortex rope and resonances are hard to detect in the acceleration in the bearings (see Figure 5) since they are a phenomena at very low frequency and the accelerometers are not the best sensors to measure those frequencies. However, another important phenomenon in terms of acceleration is detected with these accelerometers. At P/P_rated_ = 0.29, a frequency range between 2–6 times *f_f_* (4.2–12.8 Hz) is excited with considerable amplitude especially in the generator bearing. According to numerical simulations [18], some natural frequencies of the whole rotating train, including runner, shaft and generator are in this zone, and they have an important motion in the generator side. This means that for this operating point (P/P_rated_ = 0.29), those natural frequencies are excited. As it is a wide range random excitation, according to the FFT signature, the origin could be the stochastic behaviour of the fluid and the high level of turbulence in that operating point in DPL.

**Figure A1:**
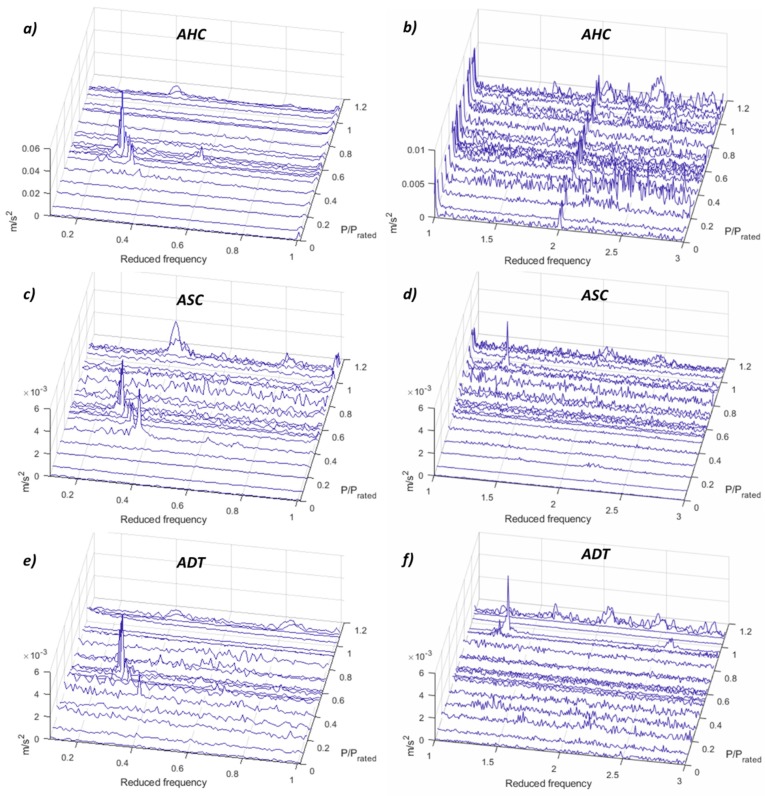
FFT waterfall of the accelerometers. (**a**) AHC 0.1–1 *f_f_*. (**b**) AHC 1–3 *f_f_*. (**c**) ASC 0.1–1 *f_f_*. (**d**) ASC 1–3 *f_f_*. (**e**) ADT 0.1–1 *f_f_*. (**f**) ADT 1–3 *f_f_*.

**Figure A2:**
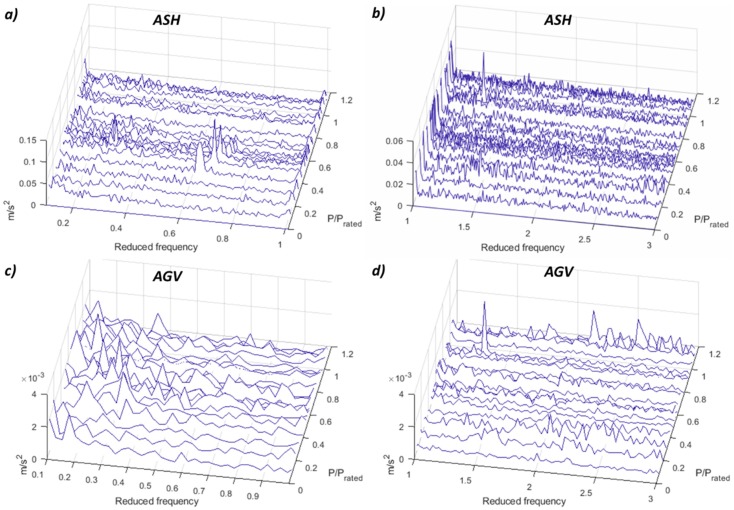
FFT waterfall of the accelerometers. (**a**) ASH 0.1–1 *f_f_*. (**b**) ASH 1–3 *f_f_*. (**c**) AGV 0.1–1 *f_f_*. (**d**) AGV 1–3 *f_f_*.

**Figure A3:**
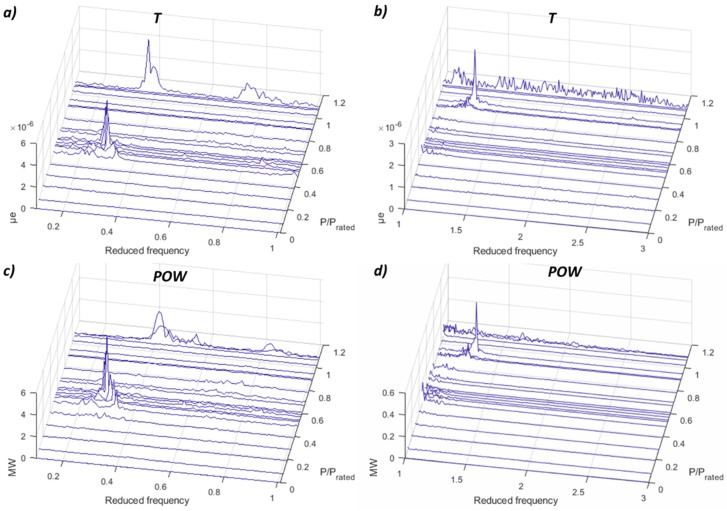
FFT waterfall of the torque (strain in the shaft) and power. (**a**) T 0.1–1 *f_f_*. (**b**) T 1–3 *f_f_*. (**c**) POW 0.1–1 *f_f_*. (**d**) POW 1–3 *f_f_*.

**Figure A4:**
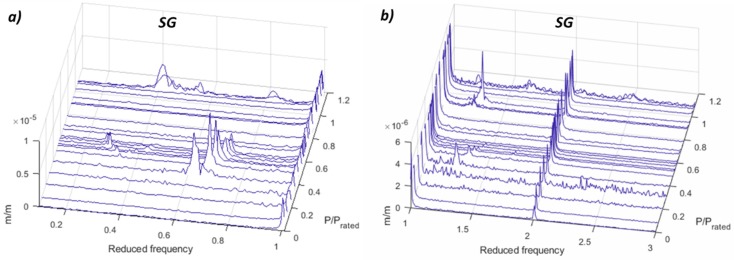
FFT Waterfall of the strain gauge in the runner. (**a**) 0.1–1 *f_f_*. (**b**) 1–3 *f_f_*.

**Figure A5:**
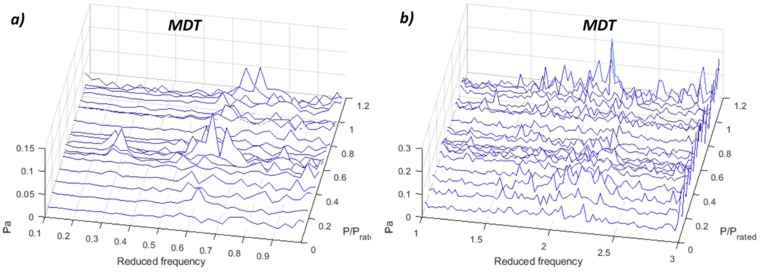
FFT waterfall of the microphone in the draft tube. (**a**) 0.1–1 *f_f_*. (**b**) 1–3 *f_f_*.

The results for the rest of the sensors are shown in Appendix A, Figure A1, Figure A2, Figure A3, Figure A4 and Figure A5. The torque and power fluctuate (see Figure A3) at the PL and HPL resonances as well as in the overload instability, as it was confirmed previously in [22]. The strain gauge in the runner (Figure A4) detects the PL vortex rope but viewed from the rotating point of view (0.6–0.8 *f_f_*) and the overload instability at the same frequency since it has mainly an axial component which is seen exactly the same from stationary or rotating points of view.

#### 5.1.2. Medium Frequency

**Figure 6:**
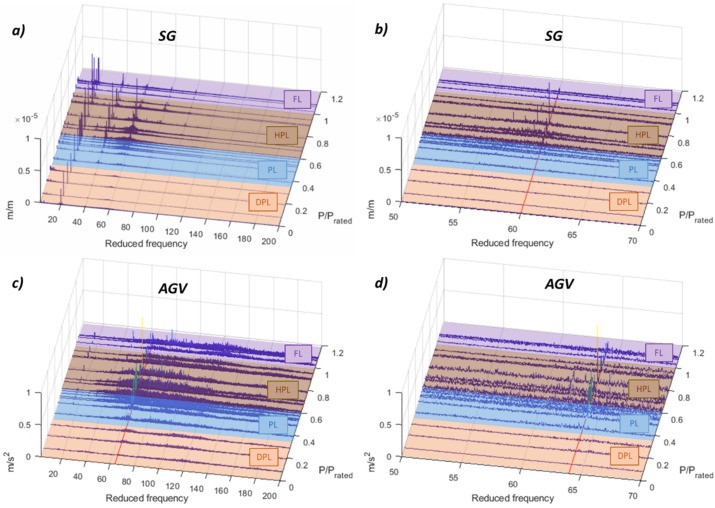
FFT waterfall of the strain gauge in the runner and the accelerometer in the guide vane. (**a**) SG 3–200 *f_f_*. (**b**) SG 50–70 *f_f_*. (**c**) AGV 3–200 *f_f_*. (**d**) AGV 50–70 *f_f_*.

In the medium frequency range, which in this case is defined from 3–200 *f_f_*, phenomena related with the RSI might be detected. For this case, in the HPL regime, a problem with the third harmonic of the RSI is detected. In the strain gauge of the runner (see Figure 6a,b), the second and third harmonic clearly increases in the HPL regime. In addition, it seems that the third harmonic of the RSI coincides with a natural frequency of the runner (this was previously studied in [17]), hence in this case the amplitude increases considerably. From the stationary point of view, this phenomenon is also clearly detected in the accelerometer in the guide vane (see Figure 6a,b), but in this case, it is the fourth harmonic of the RSI that increases considerably. This means that, according to Equation (6), the excitation shape of the interaction between the third harmonic of the RSI from the rotating point of view and the fourth harmonic of the RSI from the stationary point of view is +4. At this frequency a mode-shape of the runner with this shape was identified in [17], so a mechanical resonance of the runner at this operating point is confirmed. The reason for why the RSI changes only at the HPL regime could be due to inlet cavitation, which forms attached cavities in the inlet of the runner that change the pressure distribution in the inlet and therefore also the RSI. This inlet cavitation is confirmed with the demodulation analysis done and presented in the following sections.

**Figure 7:**
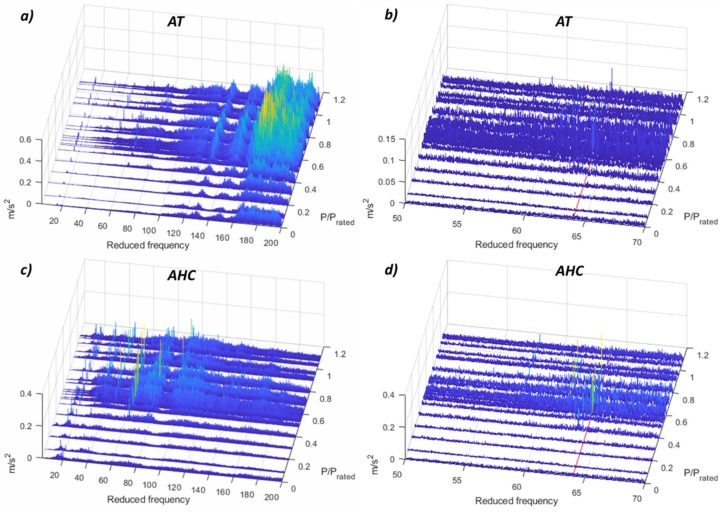
FFT Waterfall of the accelerometers in the turbine bearing and head cover. (**a**) AT 3–200 *f_f_*. (**b**) AT 50–70 *f_f_*. (**c**) AHC 3–200 *f_f_*. (**d**) AHC 50–70 *f_f_*.

**Figure A6:**
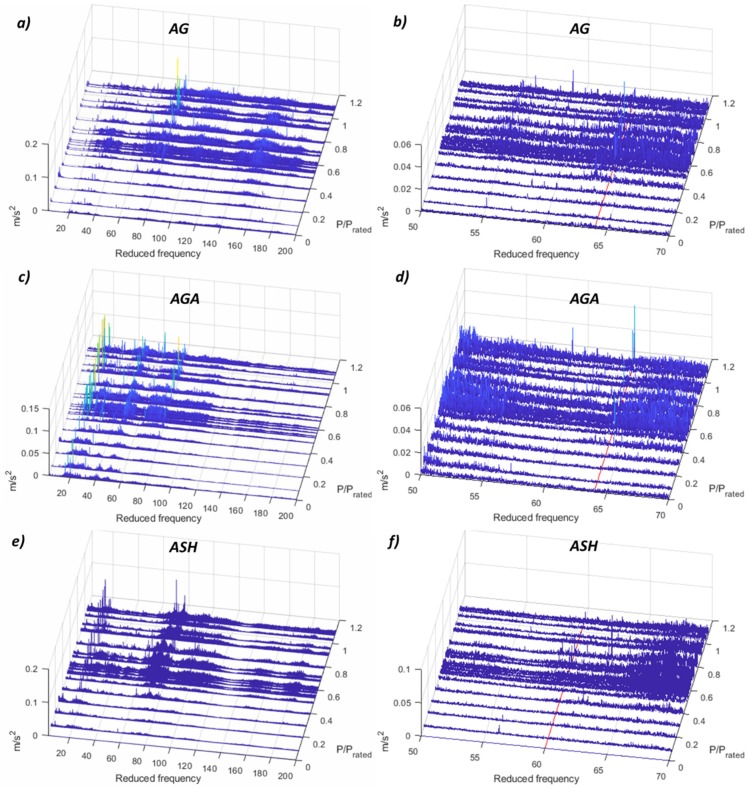
FFT waterfall of different accelerometers. (**a**) AG 3–200 *f_f_*. (**b**) AG 50–70 *f_f_*. (**c**) AGA 3–200 *f_f_*. (**d**) AGA 50–70 *f_f_*. (**e**) ASH 3–200 *f_f_*. (**f**) ASH 50–70 *f_f_*.

**Figure A7:**
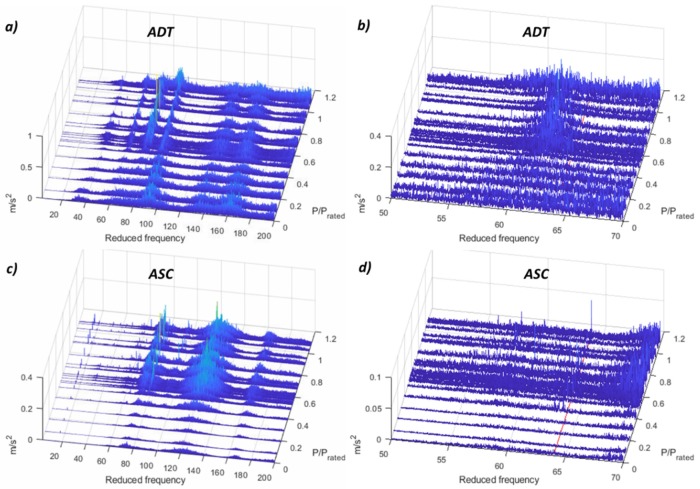
FFT waterfall of different accelerometers. (**a**) ADT 3–200 *f_f_*. **(b**) ADT 50–70 *f_f_*. (**c**) ASC 3–200 *f_f_*. (**d**) ASC 50–70 *f_f_*.

**Figure A8:**
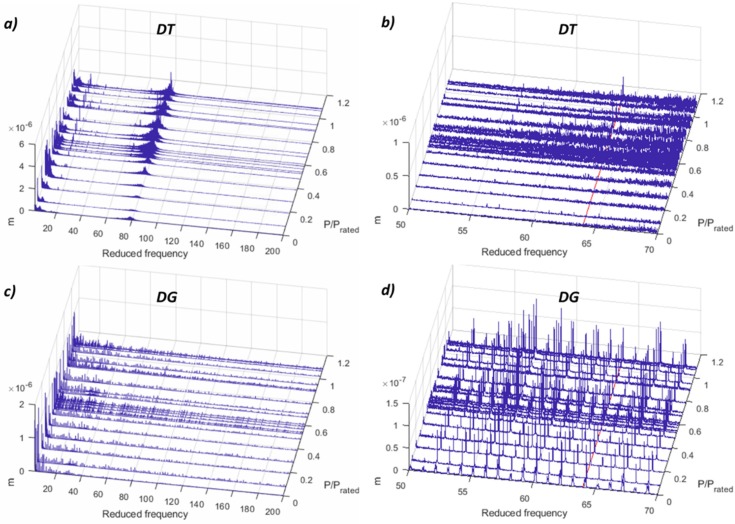
FFT saterfall of the proximity probes. (**a**) DT 3–200 *f_f_*. (**b**) DT 50–70 *f_f_*. (**c**) DG 3–200 *f_f_*. (**d**) DG 50–70 *f_f_*.

**Figure A9:**
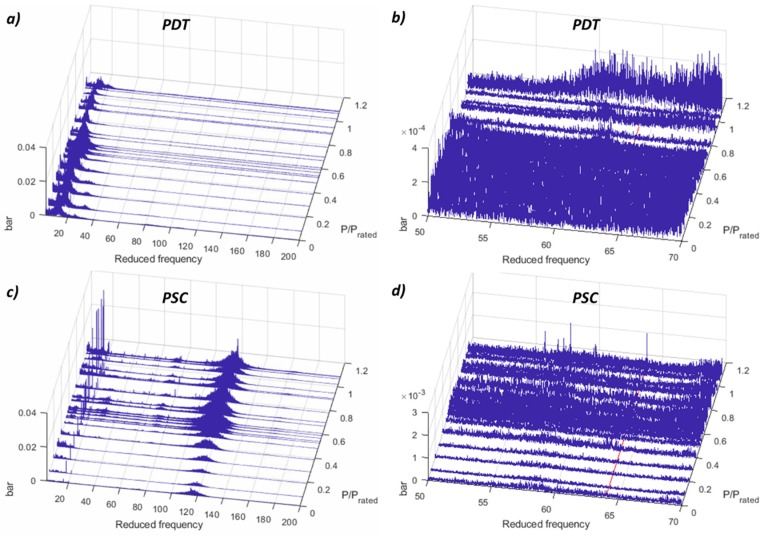
FFT waterfall of the pressure sensors. (**a**) PDT 3–200 *f_f_*. (**b**) PDT 50–70 *f_f_*. (**c**) PSC 3–200 *f_f_*. (**d**) PSC 50–70 *f_f_*.

**Figure A10:**
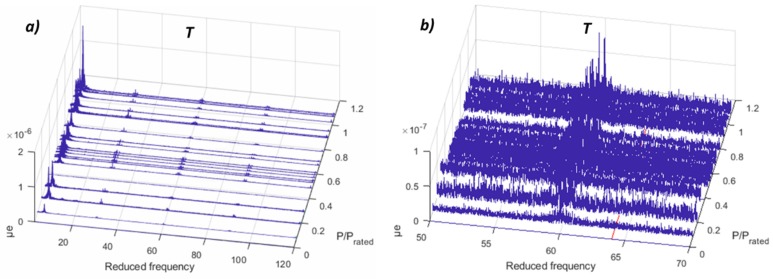
FFT waterfall of the torque. (**a**) T 3–200 *f_f_*. (**b**) T 50–70 *f_f_*.

**Figure A11:**
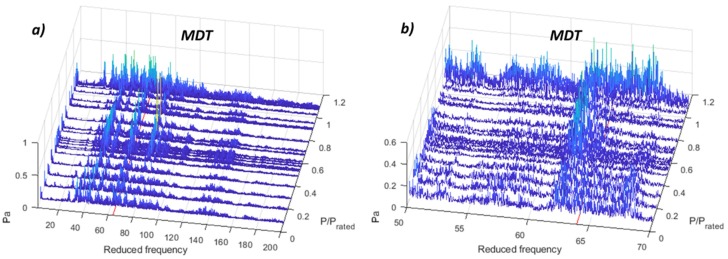
FFT waterfall of the microphone in the draft tube. (**a**) MDT 3–200 *f_f_*. (**b**) MDT 50–70 *f_f_*.

Apart from the accelerometer in the guide vane, the best sensors to detect this runner resonance from the stationary point of view are the accelerometers in THE head cover and in the turbine bearing (see Figure 7). The accelerometer in the turbine bearing detects also high amplitudes of wide frequency ranges at about 150–200 *f_f_*, which could be also related to cavitation. The rest of the sensors do not detect this phenomenon. The FFT waterfalls are shown in Appendix A Figure A6, Figure A7, Figure A8, Figure A9, Figure A10 and Figure A11.

### 5.2. RMS

**Figure 8:**
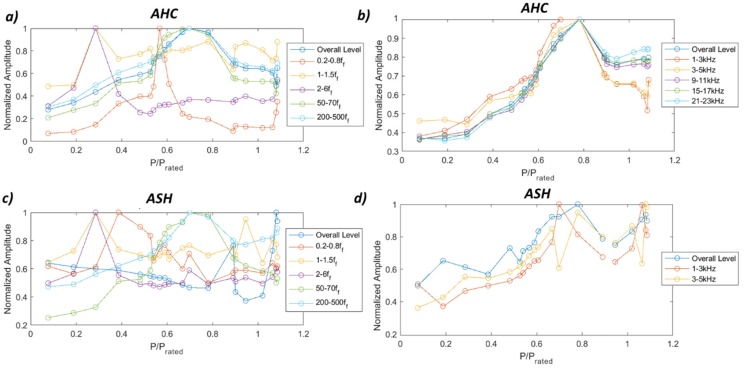
Root mean square (RMS) values for the accelerometers in the head cover and in the shaft. (**a**) AHC 0.2–500 *f_f_*. (**b**) AHC 1–23 kHz. (**c**) ASH 0.2–500 *f_f_*. (**d**) ASH 1–5 kHz.

**Figure A12:**
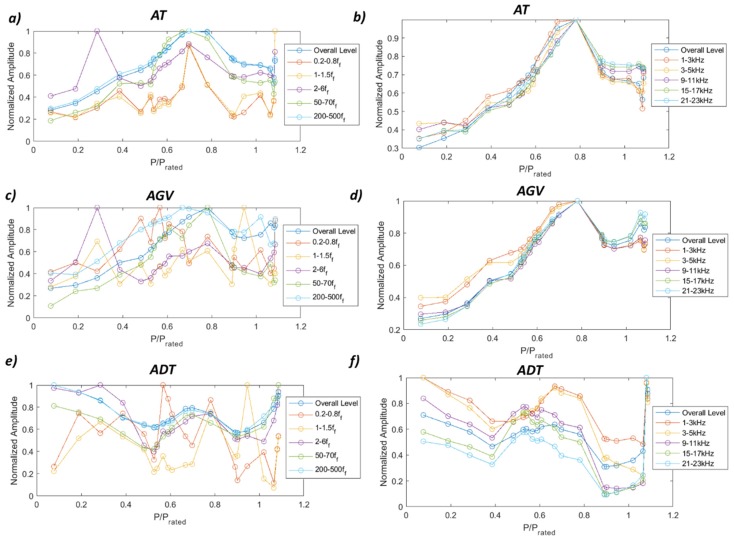
RMS values for different accelerometers. (**a**) AT 0.2–500 *f_f_*. (**b**) AT 1–23 kHz. (**c**) AGV 0.2–500 *f_f_*. (**d**) AGV 1–23 kHz. (**e**) ADT 0.2–500 *f_f_*. (**f**) ADT 1–23 kHz.

**Figure A13:**
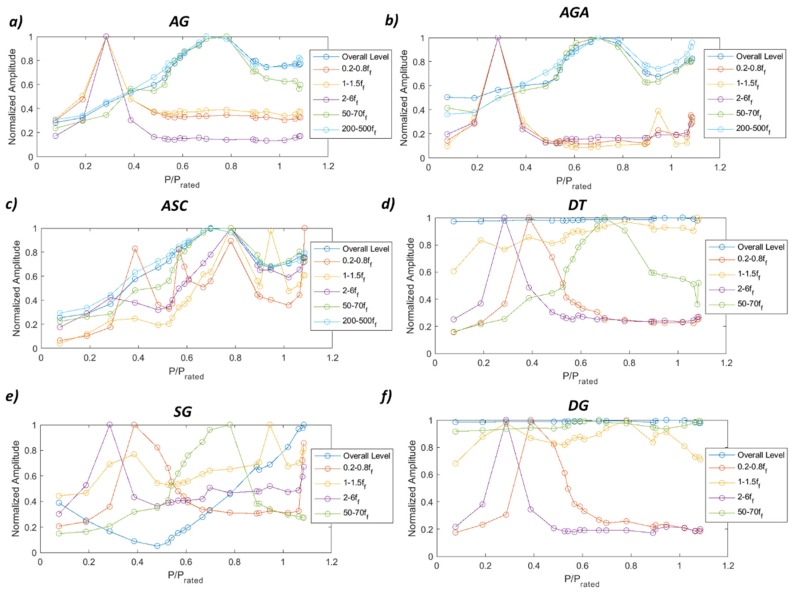
RMS values for different sensors. (**a**) AG 0.2–500 *f_f_*. (**b**) AGA 0.2–500 *f_f_*. (**c**) ASC 0.2–500 *f_f_*. (**d**) DT 0.2–70 *f_f_*. (**e**) SG 0.2–70 *f_f_*. (**f**) DG 0.2–70 *f_f_*.

**Figure A14:**
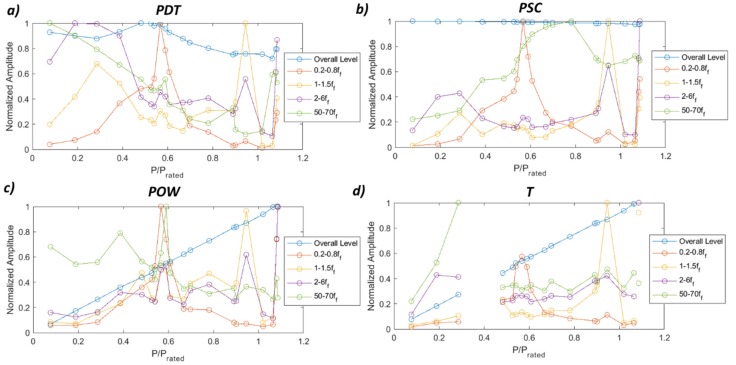
RMS values for different sensors. (**a**) PDT 0.2–70 *f_f_*. (**b**) PSC 0.2–70 *f_f_*. (**c**) POW 0.2–70 *f_f_*. (**d**) T 0.2–70 *f_f_*.

**Figure A15:**
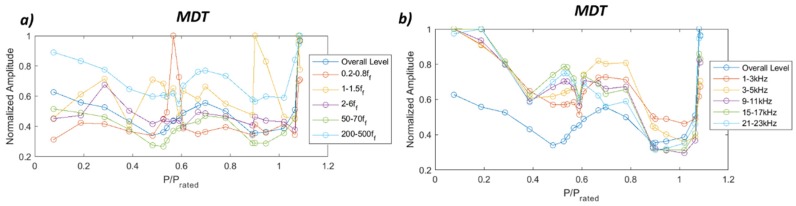
RMS values for the microphone in the draft tube. (**a**) MDT 0.2–500 *f_f_*. (**b**) MDT 1–23 kHz.

According to the results obtained with the FFT analysis, different frequency bands have been selected in order to detect the hydraulic phenomena. Those bands are 0.2–0.8 *f_f_*, 1–1.5 *f_f_*, 2–6 *f_f_*, 50–70 *f_f_*, 200–500 *f_f_*, and high-frequency bands every 2 kHz from 1 kHz until 23 kHz. Figure 8 shows the results obtained for the two accelerometers that are able to detect more phenomena with RMS values, the accelerometer in the head cover and the accelerometer in the shaft. The accelerometer in the head cover clearly detects the PL resonance and the overload instability in the frequency band of 0.2–0.8 *f_f_*, the excitation of the rotating train natural frequency in the frequency band 2–6 *f_f_*, the excitation of the runner natural frequency in the frequency band 50–70 *f_f_* and the cavitation in the HPL regime in all high-frequency bands. The accelerometer in the shaft presents similar results with the difference that it is able to detect the asynchronous component of the vortex rope in the band 0.2–0.8 *f_f_* and not the PL resonance. The results for the rest of the sensors are shown in Appendix B Figure A12, Figure A13, Figure A14 and Figure A15 and in Table 2.

### 5.3. Demodulation

The demodulation technique has been only applied to those sensors in the stationary frame with the highest acquisition frequency (ADT, AGV, AHC, AT and MDT, see Table 1) and to the accelerometer in the rotating frame (ASH). This technique is based on the hypothesis that the high frequency of the vibration and sound is modulated with the most important low-frequency phenomena. In that way, after applying the demodulation technique, it is expected to find the periodic hydraulic phenomena. Those frequencies are normally the frequency of the vortex rope or hydraulic resonances when those phenomena are taking place in the turbine or the RSI frequencies when cavitation appears, especially for the inlet cavitation [35].

**Figure 9:**
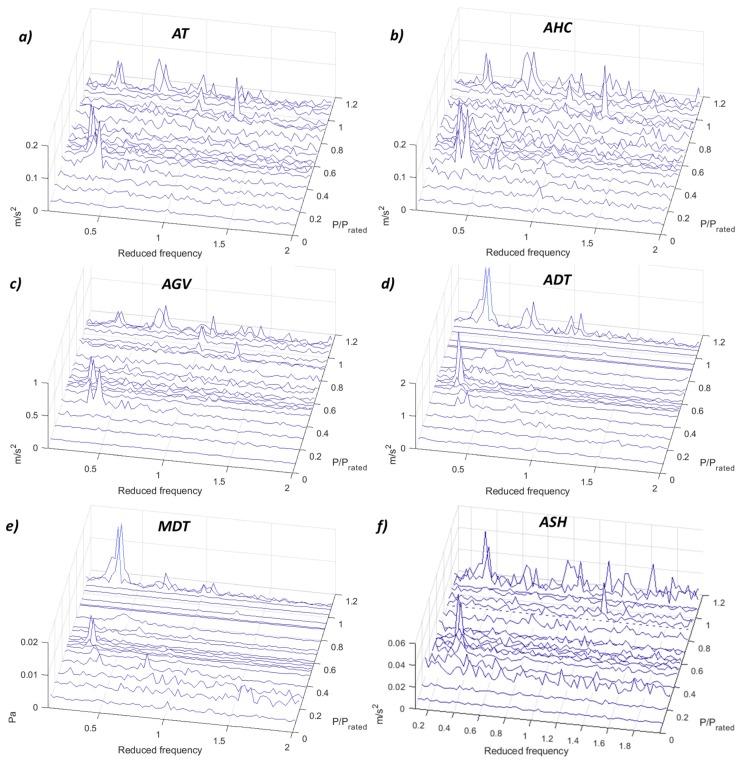
Demodulation waterfall (0.1–2 *f_f_*) of the different sensors. (**a**) AT 13–5 kHz. (**b**) AHC 13–15 kHz. (**c**) AGV 13–15 kHz. (**d**) ADT 13–15 kHz. (**e**) MDT 13–15 kHz. (**f**) ASH 5–6 kHz.

The most challenging part of applying the demodulation technique is to select the correct high-frequency band. It is not clear which is the frequency band that has to be chosen for every sensor and to detect every phenomenon. For that, different frequency ranges have been selected and compared for every sensor. Results are shown in Video S1 to Video S5 in the Supplementary Material. The best frequency range to detect the low-frequency phenomena for all the sensors and for this machine is from 13 kHz in advance. From this frequency range, the peaks of the vortex rope and the different hydraulic resonances are clearly detected, and their amplitudes are increasing at the same time than the frequency ranges. Figure 9 shows the detection of the low-frequency phenomena for the different sensors. It is observed that all of the accelerometers presented in the figure as well as the microphone in the draft tube are able to detect these phenomena. It should be noted that the demodulation of the accelerometer in the shaft (ASH) is applied within the frequency range 5–6 kHz, since it is the maximum possible for this sensor (see Table 1).

**Figure 10:**
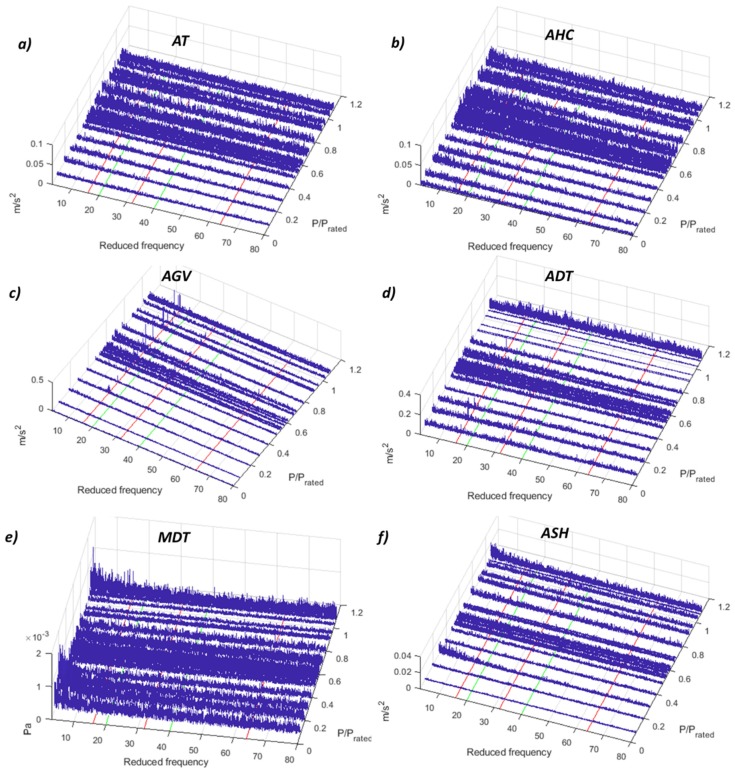
Demodulation waterfall (2–80 *f_f_*) of the different sensors. (**a**) AT 13–15 kHz. (**b**) AHC 13–15 kHz. (**c**) AGV 13–15 kHz. (**d**) ADT 13–15 kHz. (**e**) MDT 13–15 kHz. (**f**) ASH 5–6 kHz.

To detect cavitation, the demodulation technique has been used in the past [34,35]. The theory says that inlet cavitation is modulated with the RSI, hence if the RSI frequencies appear once demodulating high-frequency signals, this means that cavitation is taking place. In this case, the results for the demodulation of a high-frequency band (13–15 kHz) are shown in Figure 10. In these plots, the first three RSI frequencies from the stationary point of view are highlighted in red, and the first two from the rotating point of view in green. Only in the accelerometer located in the guide vane, the RSI frequencies are obtained after the demodulation. In the accelerometer located in the shaft (ASH), the RSI frequency viewed from the rotating frame appears for one operating point (P/P_rated_ = 0.8), which is the point that presents the highest amplitude in the demodulation obtained with the AGV. This could mean that at this operating condition the cavitation presents more erosive characteristics, as was discussed previously in [34,35].

### 5.4. Summary

To summarize all the results about the detection of the different phenomena, Table 2 is presented. In this table, all the phenomena appearing in this machine are included as well as if they are detected with every sensor used. The detection of the different phenomena has been assessed qualitatively with three different levels: not detected, poor detection and good detection. When one phenomenon is detected, it is included how it is detected, explaining at which frequency or in which frequency band is detected. In addition, the number of phenomena detected with every sensor is included in order to be able to select the best sensor to detect as many phenomena as possible.

The sensors that are able to detect more phenomena are the accelerometers in the turbine bearing, head cover, guide vanes and shaft, and the ones that detect less are the accelerometer and displacement probe located in the generator bearing. With this information the current monitoring systems can be improved and optimized using less channels, and therefore smaller amounts of data, obtaining similar results for the detection of the different phenomena.

## 6. Conclusions

The detection of the different phenomena taking place in Francis turbines have been studied. For this purpose, a large Francis turbine was selected. Different types of sensors such as accelerometers, proximity probes, strain gauges and a microphone have been located in different parts of the machine. For this Francis turbine, different phenomena have been detected in its whole operating range: vortex rope, hydraulic resonances, instabilities, cavitation and mechanical resonances of the runner and rotating train. Different techniques have been used to detect all of these phenomena. Results have been presented in a table where all the sensors and detection techniques have been compared for the different operating conditions of the Francis turbine.

FFT, RMS and demodulation techniques have been applied to the different sensors located along the machine. Every phenomenon can be detected with a different technique in every sensor. Results show that some sensors are able to detect some phenomena using non-conventional techniques, such as demodulation analysis, that are not commonly used for this purpose. The sensors that are able to detect more phenomena in this machine are the accelerometers located in the turbine bearing, head cover, guide vane and in the shaft. In those accelerometers, performing an FFT of the signal is not enough to obtain directly the information of what is happening in the machine; hence high-frequency bands need to be demodulated to obtain the origin of the excitation.

The information presented in this paper is important for monitoring hydropower units, specifically Francis turbines. The current monitoring systems are used to ensure a safe operation of the units and to perform predictive maintenance of them. However, with this study they could be improved to be used as detection systems of the different phenomena. Detecting the phenomena taking place in the unit in real-time leads to the identification of the worse operating points of the machine and therefore to optimizing the current way to operate them, reducing the maintenance costs and increasing the useful lifetime of the turbine components.

## Figures and Tables

**Table 1:** Sensors used for this study: their nomenclature, location, direction and acquisition frequency.

Type	Nomenclature	Location	Direction	Acquisition Frequency
Accelerometer	AT	Turbine bearing	Radial	65,536 Hz
Accelerometer	AG	Generator bearing	Radial	4096 Hz
Accelerometer	AGA	Thrust bearing	Axial	4096 Hz
Accelerometer	ADT	Draft tube	Radial	65,536 Hz
Accelerometer	ASC	Spiral casing	Radial	4096 Hz
Accelerometer	AHC	Head cover	Axial	65,536 Hz
Accelerometer	AGV	Guide vane	Radial	65,536 Hz
Proximity probe	DT	Turbine bearing	Radial	4096 Hz
Proximity probe	DG	Generator bearing	Radial	4096 Hz
Pressure sensor	PDT	Draft tube	Radial	4096 Hz
Pressure sensor	PSC	Draft tube	Radial	4096 Hz
Power transducer	POW	Generator	-	4096 Hz
Microphone	MDT	Draft tube	-	65,536 Hz
Strain gauge	T	Shaft	Torsional	512 Hz
Strain gauge	SG	Runner blade	-	4096 Hz
Accelerometer	ASH	Shaft	Radial	12,000 Hz

**Table 2:** Summary of the phenomena detected with every sensor and in which range they are detected.

Operating Regime	P/P_rated_	Phenomenon	Detection Technique	Sensors															
				Stationary Frame													Rotating Frame		
				Acceleration							Displacement		Pressure		Power	Sound	Torque	Strain	Acc
				AT	AG	AGA	ADT	ASC	AHC	AGV	DT	DG	PDT	PSC	POW	MDT	T	SG	ASH
Deep Part Load	0.286	Turbulence exciting rotating train natural frequency	FFT	(2–6) *f_f_*	(2–6) *f_f_*	(2–6) *f_f_*	No	No	(2–6) *f_f_*	(4–10) *f_f_*	(2–6) *f_f_*	(2–6) *f_f_*	No	No	No	No	No	(2–6) *f_f_*	No
			RMS	(2–6) *f_f_*	(2–6) *f_f_*	(2–6) *f_f_*	(2–6) *f_f_*	No	(1–6) *f_f_*	(2–6) *f_f_*	(2–6) *f_f_*	(2–6) *f_f_*	(2–6) *f_f_*	No	No	(2–6) *f_f_*	No	(2–6) *f_f_*	(1–6) *f_f_*
			Demodulation	No			No		(2–6) *f_f_*	No						No			No
Part Load	0.3874–0.6081	Vortex rope— asynchronous component	FFT	No	(0.2–0.4) *f_f_*	No	No	No	No	No	(0.2–0.4) *f_f_*	(0.2–0.4) *f_f_*	(0.2–0.4) *f_f_*	No	No	No	No	(0.6–0.8) *f_f_*	(0.6–0.8) *f_f_*
			RMS	No	No	No	No	No	No	No	(0.2–0.8) *f_f_*	(0.2–0.8) *f_f_*	No	No	No	No	No	(0.2–0.8) *f_f_*	(0.2–0.8) *f_f_*
			Demodulation	No			No		No	>19 kHz						No			No
		Vortex Rope— synchronous component	FFT	No	No	No	(0.2–0.4) *f_f_*	(0.2–0.4) *f_f_*	(0.2–0.4) *f_f_*	No	No	No	(0.2–0.4) *f_f_*	(0.2–0.4) *f_f_*	(0.2–0.4) *f_f_*	(0.2–0.4) *f_f_*	(0.2–0.4) *f_f_*	(0.6–0.8) *f_f_*	No
			RMS	No	No	No	(0.2–0.8) *f_f_*	(0.2–0.8) *f_f_*	(0.2–0.8) *f_f_*	No	No	No	(0.2–0.8) *f_f_*	(0.2–0.8) *f_f_*	(0.2–0.8) *f_f_*	(0.2–0.8) *f_f_*	(0.2–0.8) *f_f_*	No	No
			Demodulation	>13 kHz			>13 kHz		>13 kHz	No						>13 kHz			>5 kHz
	0.59	Hydraulic resonance	FFT	No	No	No	0.3 *f_f_*	0.3 *f_f_*	0.3 *f_f_*	No	No	No	0.3 *f_f_*	0.3 *f_f_*	0.3 *f_f_*	0.3 *f_f_*	0.3 *f_f_*	No	No
			RMS	No	No	No	(0.2–0.8) *f_f_*	(0.2–0.8) *f_f_*	(0.2–0.8) *f_f_*	(0.2–0.8) *f_f_*	No	No	(0.2–0.8) *f_f_*	(0.2–0.8) *f_f_*	(0.2–0.8) *f_f_*	(0.2–0.8) *f_f_*	(0.2–0.8) *f_f_*	No	No
			Demodulation	>13 kHz			>13 kHz		>13 kHz	>13 kHz						>13 kHz			>5 kHz
High Part Load	0.5405–0.8919	Cavitation	FFT	No	No	No	No	No	No	(3–200) *f_f_*	No	No	No	No	No	No	No	No	No
			RMS	>200 *f_f_*	>200 *f_f_*	>200 *f_f_*	>200 *f_f_*	>200 *f_f_*	>200 *f_f_*	>200 *f_f_*	No	No	No	No	No	No	No	No	(200–500) *f_f_*
			Demodulation	No			No		No	>13 kHz						No			>5 kHz
	0.7815	Runner resonance	FFT	f_b,4_	f_b,4_	f_b,4_	(60–65) *f_f_*	No	f_b,4_	f_b,4_	No	No	No	No	No	No	No	f_v,3_	No
			RMS	(50–70) *f_f_*	(50–70) *f_f_*	(50–70) *f_f_*	No	(50–70) *f_f_*	(50–70) *f_f_*	(50–70) *f_f_*	(50–70) *f_f_*	No	No	(50–70) *f_f_*	No	No	No	(50–70) *f_f_*	(50–70) *f_f_*
			Demodulation	No			No		No	>13 kHz						No			No
	0.9459	Hydraulic resonance	FFT	1.3 *f_f_*	No	1.3 *f_f_*	1.3 *f_f_*	1.3 *f_f_*	No	1.3 *f_f_*	1.3 *f_f_*	No	1.3 *f_f_*	1.3 *f_f_*	1.3 *f_f_*	No	1.3 *f_f_*	1.3 *f_f_*	1.3 *f_f_*
			RMS	No	No	(1–1.5) *f_f_*	(1–1.5) *f_f_*	(1–1.5) *f_f_*	No	(1–1.5) *f_f_*	No	No	(1–1.5) *f_f_*	(1–1.5) *f_f_*	(1–1.5) *f_f_*	(1–1.5) *f_f_*	(1–1.5) *f_f_*	(1–1.5) *f_f_*	(1–1.5) *f_f_*
			Demodulation	>13 kHz			>13 kHz		>13 kHz	>13 kHz	No	No	No	No	No	>13 kHz	No	No	>5 kHz
Overload	1.0856	Overload instability	FFT	No	No	No	0.4 *f_f_*	0.4 *f_f_*	0.4 *f_f_*	No	No	No	0.4 *f_f_*	0.4 *f_f_*	0.4 *f_f_*	No	0.4 *f_f_*	0.4 *f_f_*	No
			RMS	No	No	0.2–6 f_f_	No	0.2–0.8 *f_f_*	0.2–0.8 *f_f_*	0.2–0.8 *f_f_*	No	No	0.2–0.8 *f_f_*	0.2–0.8 *f_f_*	0.2–0.8 *f_f_*	Overall	0.2–0.8 *f_f_*	0.2–0.8 *f_f_*	Overall
			Demodulation	>13 kHz			>13 kHz		>13 kHz	>13 kHz						>13 kHz			>5 kHz

Number of phenomena detected (only the phenomena with good detection are counted)			FFT	1	1	2	3	3	3	4	2	2	4	5	4	0	4	4	0
			RMS	3	3	2	2	4	5	3	3	2	4	4	4	4	4	5	4
			Demodulation	4	-	-	4	-	5	6	-	-	-	-	-	4	-	-	5
			Total	7	3	4	5	5	7	7	3	2	4	5	4	4	4	5	7
																	
						Not detected			Poor detection			Good detection			Not applicable				

## Supplementary Materials

The following are available online at https://www.mdpi.com/1424-8220/19/18/4053/s1, Video S1: Demodulation of AT in different frequency bands. Video S2: Demodulation of AHC in different frequency bands. Video S3: Demodulation of AGV in different frequency bands. Video S4: Demodulation of ADT in different frequency bands. Video S5: Demodulation of MDT in different frequency bands.

## References

[1] Gaudard L., Romerio F. (2014). The future of hydropower in Europe: Interconnecting climate, markets and policies. Environ. Sci. Policy.

[2] Bélanger C., Gagnon L. (2002). Adding wind energy to hydropower. Energy Policy.

[3] Pereira J.G., Andolfatto L., Avellan F. (2018). Monitoring a Francis turbine operating conditions. Flow Meas. Instrum..

[4] IEC 60193 (1999). Hydraulic Turbines, Storage Pumps and Pump-Turbines Model Acceptance Tests.

[5] Presas A., Valentín D., Egusquiza M., Valero C., Egusquiza E. (2018). Sensor-Based Optimized Control of the Full Load Instability in Large Hydraulic Turbines. Sensors.

[6] Presas A., Egusquiza E., Valero C. (2017). Detection and analysis of part load and full load instabilities in a real Francis turbine prototype. J. Physics Conf. Ser..

[7] Favrel A., Junior J.G.P., Landry C., Alligné S., Andolfatto L., Nicolet C., Avellan F. (2019). Prediction of hydro-acoustic resonances in hydropower plants by a new approach based on the concept of swirl number. J. Hydraul. Res..

[8] Presas A., Valentin D., Egusquiza E., Valero C., Seidel U. (2015). On the detection of natural frequencies and mode shapes of submerged rotating disk-like structures from the casing. Mech. Syst. Signal Process..

[9] Bossio M., Valentín D., Presas A., Martin D.R., Egusquiza E., Valero C., Egusquiza M. (2017). Numerical study on the influence of acoustic natural frequencies on the dynamic behaviour of submerged and confined disk-like structures. J. Fluids Struct..

[10] Valero C., Egusquiza M., Egusquiza E., Presas A., Valentin D., Bossio M. (2017). Extension of Operating Range in Pump-Turbines. Influence of Head and Load. Energies.

[11] Egusquiza M., Egusquiza E., Valero C., Presas A., Valentín D., Bossio M. (2018). Advanced condition monitoring of Pelton turbines. Measurement.

[12] Egusquiza E., Valero C., Valentin D., Presas A., Rodriguez C.G. (2015). Condition monitoring of pump-turbines. New challenges. Measurement.

[13] Valero C., Egusquiza E., Presas A., Valentín D., Bossio M. (2017). Condition monitoring of a prototype turbine. Description of the system and main results. J. Physics Conf. Ser..

[14] Egusquiza E., Valero C., Huang X., Jou E., Guardo A., Rodriguez C. (2012). Failure investigation of a large pump-turbine runner. Eng. Fail. Anal..

[15] Egusquiza M., Egusquiza E., Valentin D., Valero C., Presas A. (2017). Failure investigation of a Pelton turbine runner. Eng. Fail. Anal..

[16] Zhang M., Valentín D., Valero C., Egusquiza M., Egusquiza E. (2019). Failure investigation of a Kaplan turbine blade. Eng. Fail. Anal..

[17] Valentín D., Presas A., Bossio M., Egusquiza M., Egusquiza E., Valero C. (2018). Feasibility of Detecting Natural Frequencies of Hydraulic Turbines While in Operation, Using Strain Gauges. Sensors.

[18] Valentín D., Ramos D., Bossio M., Presas A., Egusquiza E., Valero C. (2016). Influence of the boundary conditions on the natural frequencies of a Francis turbine. IOP Conf. Series: Earth Environ. Sci..

[19] Yamamoto K., Müller A., Favrel A., Landry C., Avellan F. Flow characteristics and influence associated with inter-blade cavitation vortices at deep part load operations of a Francis turbine. Proceedings of the Hyperbole Conference.

[20] Yamamoto K., Favrel A., Avellan F., Müller A. (2017). Experimental evidence of inter-blade cavitation vortex development in Francis turbines at deep part load condition. Exp. Fluids.

[21] Rheingans W.J. (1940). Power swings in hydroelectric power plants. Trans. ASME.

[22] Valentín D., Presas A., Egusquiza E., Valero C., Egusquiza M., Bossio M. (2017). Power Swing Generated in Francis Turbines by Part Load and Overload Instabilities. Energies.

[23] Favrel A., Müller A., Landry C., Yamamoto K., Avellan F. (2015). Study of the vortex-induced pressure excitation source in a Francis turbine draft tube by particle image velocimetry. Exp. Fluids.

[24] Favrel A., Müller A., Landry C., Gomes J., Yamamoto K., Avellan F. Dynamics of the precessing vortex rope and its interaction with the system at Francis turbines part load operating conditions. Proceedings of the Hyperbole Conference.

[25] Favrel A., Müller A., Landry C., Yamamoto K., Avellan F. (2016). LDV survey of cavitation and resonance effect on the precessing vortex rope dynamics in the draft tube of Francis turbines. Exp. Fluids.

[26] Favrel A., Junior J.G.P., Landry C., Müller A., Yamaishi K., Avellan F. (2019). Dynamic modal analysis during reduced scale model tests of hydraulic turbines for hydro-acoustic characterization of cavitation flows. Mech. Syst. Signal Process..

[27] Ruchonnet N., Nicolet C., Avellan F. One-dimensional modeling of rotor stator interaction in Francis pump-turbine. Proceedings of the Proceedings of the 23rd IAHR Symposium on Hydraulic Machinery and Systems.

[28] Tanaka H. (2011). Vibration Behavior and Dynamic Stress of Runners of Very High Head Reversible Pump-turbines. Int. J. Fluid Mach. Syst..

[29] Presas A., Egusquiza E., Valero C., Valentín D., Seidel U. (2014). Feasibility of Using PZT Actuators to Study the Dynamic Behavior of a Rotating Disk due to Rotor-Stator Interaction. Sensors.

[30] Müller A., Favrel A., Landry C., Avellan F. (2017). Fluid–structure interaction mechanisms leading to dangerous power swings in Francis turbines at full load. J. Fluids Struct..

[31] Müller A., Favrel A., Landry C., Yamamoto K., Avellan F. (2014). On the physical mechanisms governing self-excited pressure surge in Francis turbines. IOP Conf. Ser. Earth Environ. Sci..

[32] Müller A., Bullani A., Dreyer M., Roth S., Favrel A., Landry C., Avellan F. (2012). Interaction of a pulsating vortex rope with the local velocity field in a Francis turbine draft tube. IOP Conf. Ser. Earth Environ. Sci..

[33] Hyperbole Project HYdropower Plants PERformance and flexiBle Operation Towards Lean integration of New Renewable Energies. https://hyperbole.epfl.ch.

[34] Escaler X., Egusquiza E., Farhat M., Avellan F., Coussirat M. (2006). Detection of cavitation in hydraulic turbines. Mech. Syst. Signal Process..

[35] Valentín D., Presas A., Egusquiza M., Valero C., Egusquiza E. (2018). Transmission of High Frequency Vibrations in Rotating Systems. Application to Cavitation Detection in Hydraulic Turbines. Appl. Sci..

